# Evaluation of High-Resolution Mass Spectrometry for the Quantitative Analysis of Mycotoxins in Complex Feed Matrices

**DOI:** 10.3390/toxins11090531

**Published:** 2019-09-12

**Authors:** Tolke Jensen, Marthe de Boevre, Nils Preußke, Sarah de Saeger, Tim Birr, Joseph-Alexander Verreet, Frank D. Sönnichsen

**Affiliations:** 1 Institute of Phytopathology, Christian-Albrechts-Universität Kiel, Hermann-Rodewald-Strasse 9, 24118 Kiel, Germany; t.birr@phytomed.uni-kiel.de (T.B.); javerreet@phytomed.uni-kiel.de (J.-A.V.); 2 Centre of Excellence in Mycotoxicology and Public Health, Ghent University, Ottergemsesteenweg 460, 9000 Ghent, Belgium; Marthe.DeBoevre@UGent.be (M.d.B.); Sarah.DeSaeger@UGent.be (S.d.S.); 3 Otto Diels Institute for Organic Chemistry, Christian-Albrechts-Universität Kiel, Otto-Hahn-Platz 4, 24118 Kiel, Germany; npreusske@oc.uni-kiel.de (N.P.); fsoennichsen@oc.uni-kiel.de (F.D.S.)

**Keywords:** *Fusarium*, validation, forage maize, maize silage, LC-HRMS, Orbitrap^TM^

## Abstract

The selective and sensitive analysis of mycotoxins in highly complex feed matrices is a great challenge. In this study, the suitability of Orbitrap^TM^-based high-resolution mass spectrometry (HRMS) for routine mycotoxin analysis in complex feeds was demonstrated by the successful validation of a full MS/data-dependent MS/MS acquisition method for the quantitative determination of eight *Fusarium* mycotoxins in forage maize and maize silage according to the Commission Decision 2002/657/EC. The required resolving power for accurate mass assignments (<5 ppm) was determined as 35,000 full width at half maximum (FWHM) and 70,000 FWHM for forage maize and maize silage, respectively. The recovery (R_A_), intra-day precision (RSD_r_), and inter-day precision (RSD_R_) of measurements were in the range of 94 to 108%, 2 to 16%, and 2 to 12%, whereas the decision limit (CCα) and the detection capability (CCβ) varied from 11 to 88 µg/kg and 20 to 141 µg/kg, respectively. A set of naturally contaminated forage maize and maize silage samples collected in northern Germany in 2017 was analyzed to confirm the applicability of the HRMS method to real samples. At least four *Fusarium* mycotoxins were quantified in each sample, highlighting the frequent co-occurrence of mycotoxins in feed.

## 1. Introduction

The presence of mycotoxins in agricultural products is of increasing global concern for both food and feed safety [1]. Mycotoxins are a large group of toxic secondary metabolites mainly produced by filamentous fungi of the genera *Aspergillus*, *Penicillium*, and *Fusarium*. These secondary metabolites exert a diverse range of actions, including hepatotoxic, estrogenic, carcinogenic, mutagenic, and nephrotoxic effects [2,3]. Consequently, specific regulations or guidelines have been established related to mycotoxins in approximately 100 countries [4]. To monitor the presence of mycotoxins in food and feed, reliable analytical methods are needed [5].

The technique mostly utilized for qualitative and quantitative mycotoxin analysis relies on triple quadrupole tandem mass spectrometry. Operating in multiple reaction monitoring (MRM), the technique provides both selectivity and sensitivity [1]. Nevertheless, triple quadrupole mass analyzers show some limitations due to the acquisition mode. One drawback is the extensive and time-consuming compound-depending optimization of the acquisition parameters [6]. Another major limitation is the inability to perform retrospective data analysis [7]. For these reasons, high-resolution mass spectrometry (HRMS) offers a promising alternative. The key advantage of HRMS-based approaches lies in the acquisition of high-resolution full scan MS data that facilitates a retrospective data analysis of non-target compounds without the re-injection of samples [8]. HRMS has mainly been used for research purposes, e.g., the structural elucidation of drugs or unknown contaminants [9,10]. In the last few years, initial HRMS applications based on Orbitrap^TM^ technology focused on the quantitative analysis of mycotoxins in relatively non-complex food matrices such as wheat, corn, or barley flour [5,11,12,13,14,15,16]. However, information on the applicability of HRMS for the quantitative analysis of mycotoxins in highly complex matrices remains limited, especially regarding the detection capabilities.

The detection and quantification of mycotoxins in complex matrices (a high number of interfering matrix components relative to the analytes) is generally a great challenge [17,18]. In particular, cattle feed such as forage maize is regarded as a ‘difficult’ matrix because whole maize plants are harvested. Thus, the matrix contains not only ingredients originating from maize kernels, but also components of the vegetative part of the maize plant, e.g., chlorophyll, carotenoids, lignin, and waxes [18,19]. Further, forage maize is often ensiled in temperate regions of the world to conserve high-quality feed for winter [20]. Ensiling is based on natural fermentation whereby lactic acid bacteria metabolize carbohydrates to organic acids. As a result, the pH decreases to a level at which undesirable microorganisms are inhibited [21]. Consequently, the ensiled forage matrix ‘maize silage’ additionally contains various products of the fermentation process.

The aim of the present study was to investigate the suitability of Orbitrap^TM^-based HRMS for the quantitative analysis of mycotoxins in complex feeds, using forage maize and maize silage as representative feed matrices of high complexity. A selection of *Fusarium* mycotoxins with different physicochemical properties was included in this application based on their frequency of occurrence under the environmental conditions in northern and central Europe.

## 2. Results and Discussion

### 2.1. LC Optimization

**Figure 1:**
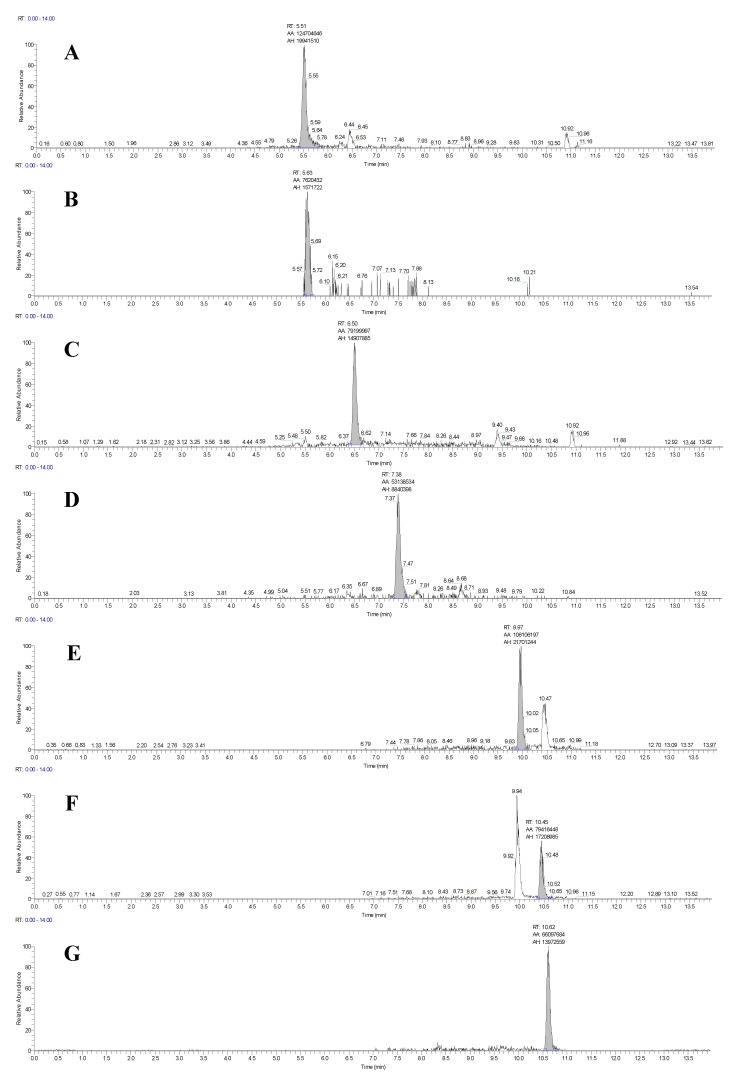
Liquid chromatography coupled to high-resolution mass spectrometry (LC-HRMS) chromatograms (Full MS) of a maize silage sample spiked at the cutoff level using a mass extraction window of ± 5 ppm, (**a**) deoxynivalenol at 400 µg/kg; (**b**) deoxynivalenol-3-glucoside at 400 µg/kg; (**c**) deepoxy-deoxynivalenol at 100 µg/kg; (**d**) 3+15-acetyl-deoxynivalenol at 100 µg/kg; (**e**) β-zearalenol at 400 µg/kg; (**f**) α-zearalenol at 500 µg/kg; and (**g**) zearalenone at 200 µg/kg.

To optimize the chromatographic separation of the mycotoxins, experiments using acetonitrile and methanol as the organic phase with different concentrations of formic acid and ammonium acetate as well as tests with different flow rates were performed (data not shown). The best peak shapes and highest peak intensities were obtained at a flow rate of 0.3 mL/min using methanol and water as mobile phases, both containing 0.1% formic acid and 5 mM ammonium acetate. LC-HRMS chromatograms of a maize silage sample acquired under these conditions and spiked at the cutoff level for each mycotoxin are shown in Figure 1. The method provides an excellent separation of the mycotoxins; even the isomers α-zearalenol (α-ZEL) and β-zearalenol (β-ZEL) were successfully baseline separated. The only exceptions were the isomers 3-acetyl-deoxynivalenol (3-AcDON) and 15-acetyl-deoxynivalenol (15-AcDON); for these, the sum of isomers was determined and reported as commonly done in the literature [22]. Interfering peaks close to the retention times of the mycotoxins as well as peak tailing have not been observed.

Inter-sample carry-over is often a significant problem in chromatographic methods of complex feed matrices. Rasmussen et al. [19] employed three time-consuming post-run cleaning steps (with acetonitrile, methanol, and water) after the injection of maize silage extracts to prevent matrix and mycotoxin accumulation on the column. To evaluate the inter-sample carry-over, blank samples containing water were injected after the matrix-matched calibration samples. A sample carry-over was not detected under any circumstances. Therefore, clean-up steps were omitted in the chromatographic method.

### 2.2. Selection of Ionization Mode

**Figure 2:**
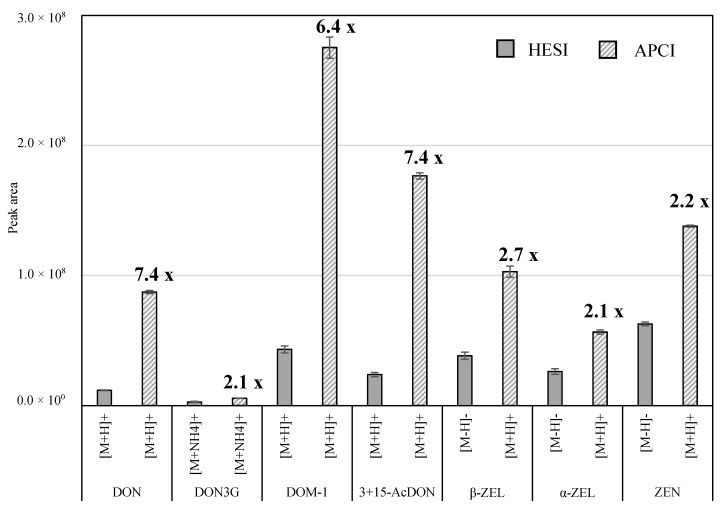
Signal intensities (means ± SD) of spiked mycotoxins in maize silage using the heated electrospray ionization (HESI) and the atmospheric pressure chemical ionization (APCI) interface (*n* = 3). The spiking level for each mycotoxin was 400 µg/kg. The precursor ion with the highest signal intensity in the spectra was chosen for the comparison. The increase of signal intensities using the APCI mode compared to the HESI mode is given as numbers above the bars. DON = deoxynivalenol; DON3G = deoxynivalenol-3-glucoside; DOM-1 = deepoxy-deoxynivalenol; 3-AcDON = 3-acetyl-deoxynivalenol; 15-AcDON = 15-acetyl-deoxynivalenol; β-ZEL = β-zearalenol; α-ZEL = α-zearalenol; ZEN = zearalenone.

The majority of published studies employed (heated) electrospray ionization ((H)ESI) as the ionization technique for the determination of mycotoxins. For example, Malysheva et al. [23] and Biselli and Hummert [24] received higher signal intensities using the ESI interface with matrix-free samples. However, Zachariasova et al. [5] reported an improved detection of multiple mycotoxins in beer using atmospheric pressure chemical ionization (APCI). Therefore, the performance of both ionization techniques was assessed in this study. For this, LC-HRMS chromatograms of spiked maize silage were generated in positive and negative ionization mode using both interfaces. For the comparison, the precursor ion with the highest intensity in the spectra was chosen for each mycotoxin (Figure 2). All *Fusarium* mycotoxins showed better ionization efficiency under APCI conditions in contrast to HESI (Figure 2). The responses achieved using APCI were 2.1 to 7.4 times higher. Due to the greater sensitivity, the APCI interface was used for the further method development. Particularly advantageous is that for all precursors, the highest intensities were obtained in the positive ion mode, so that polarity switching or a second detection run in negative ion mode could be omitted in the method.

### 2.3. Resolving Power Requirements

In HRMS analysis, selectivity considerably depends on the width of the mass extraction window (MEW) used in data processing. Narrowing the MEW eliminates interfering ions with masses outside the intended range, and thus also increases the quantitative performance [25]. The use of a narrow MEW requires a correct mass assignment, i.e., an adequate separation of analyte ions from background matrix interferences with the same nominal mass. An incorrect mass assignment and thus a poor mass accuracy can occur using an insufficient resolving power setting of the mass spectrometer [26]. Kellmann et al. [17] investigated the mass resolving power needed for the analysis of compound feed for horses (mixture of cereals), which is a matrix far less complex than those considered in the present study. As only limited information regarding the minimum required resolving power for precise mass assignments in complex feed matrices is available in the literature, this aspect was investigated in the present study. The accuracy of mass assignment was studied in full scan MS at three concentration levels and four resolving power settings (17,500, 35,000, 70,000 and 140,000 full width at half maximum (FWHM)) for both matrices, forage maize and maize silage (Table 1).

The mass resolving power setting of 17,500 FWHM resulted in high mass deviations, irrespective of the matrix. Even at the highest concentration level, mass deviations of more than 10 ppm were observed. Using the MEW screening setting of ± 5 ppm set in the guidance document on the identification of mycotoxins in food and feed (SANTE 12089/2016) [27] would consequently result in the non-detection (false negatives) of a number of mycotoxins, and thus in an erroneous quantification. With increasing resolving power, mass accuracies considerably improved. In the case of forage maize, a resolving power of 35,000 FWHM resulted in adequate mass accuracies (<5 ppm) in all cases, irrespective the concentration level. However, a resolving power of 35,000 FHWM was insufficient in case of maize silage due to high mass deviations (>10 ppm) at the lowest concentration level (Table 1). The different minimal resolving power settings for the two matrices may be due to the alteration of the matrix during the fermentation process. In contrast to forage maize, maize silage additionally contains a variety of products released by enzyme-catalyzed reactions of plant and microbial origin during the fermentation process [18]. For example, ensiled forages often contain higher amounts of free amino acids and peptides, as well as higher levels of monosaccharaides released from polysaccharides or complex oligosaccharides [28]. The higher amount of matrix compounds increases the risk of co-eluting interfering ions with similar exact masses as the mycotoxins. To baseline separate mycotoxins from those interfering ions, especially at low concentration levels, a higher resolving power (70,000 FWHM) was essential in case of maize silage. The mass accuracies between the intermediate (70,000 FHWM) and the highest resolving power setting (140,000 FHWM) were observed not to differ, which highlights that the latter resolving power is not required for accurate mass assignments in complex animal feed matrices. As a high resolving power results in longer scanning times and further in a lower number of data points per chromatographic peak, difficulties for quantification may arise [29]. Due to excellent mass accuracies, especially at low concentration levels and the facts given above, a resolving power of 70,000 FWHM was selected in the final method for both matrices. This setting enabled the use of the required MEW setting of ± 5 ppm in data processing [27].

Although the number of mycotoxins considered in the present study was relatively limited, the results clearly underline that the mass resolving power requirement highly depends on the complexity of the matrix and the concentration level of the analytes, which is in agreement with recent studies [17,26]. However, this is to our knowledge the first report showing that the processing of raw materials can result in a significant change in the required resolving power setting for accurate measurements. In recent years, the interest regarding the fate of mycotoxins during processing (e.g., baking, brewing, cooking, fermentation) is steadily growing. Based on the results of the present study, research on different processed foods or feeds using HRMS should carefully select the resolving power fit-for-purposes at the beginning of method development in order to guarantee a high selectivity and thus an excellent quantitative performance.

### 2.4. Validation of the HRMS Method

To evaluate the applicability of HRMS for routine mycotoxin analysis in complex feeds, a method for the quantitative determination of mycotoxins in forage maize and maize silage has been validated according to the Commission Decision 2002/657/EC [30]. Performance characteristics such as linearity, apparent recovery, intra-day precision, inter-day precision, specificity, measurement uncertainty, decision limit, and detection capability were investigated.

The recovery, precision, and measurement uncertainty of the developed method were determined at five concentration levels. For clarity, only the results of the lowest, medium, and highest concentration levels are given in Table 2. The recoveries (R_A_) for all mycotoxins ranged between 94 and 108%, thus fulfilling the strict requirements (80–110%) of the Commission Decision 2002/657/EC [30]. In comparison to a recently published LC-MS/MS method for the quantification of mycotoxins in maize silage [18], the recoveries achieved in our study are overall comparable and noticeably better with respect to the recovery of the polar mycotoxin deoxynivalenol-3-glucoside (DON3G). The precision of the method was investigated by means of two parameters: intra-day precision (RSD_r_) and inter-day precision (RSD_R_). The RSD_r_ and RSD_R_ values (Table 2) ranged from 2 to 16% and from 2 to 12%, respectively. Thus, all the precision values fall within the accepted range of the Commission Decision 2002/657/EC [30].

For the accurate interpretation of measurements of unknown samples, knowledge of the uncertainty of the measured results is essential [31]. Therefore, the measurement uncertainty (U) was calculated for each mycotoxin. As shown in Table 2, the U values ranged between 7 and 42%. The highest value was obtained for DON3G in maize silage due to high precision values. Overall, the results are comparable with those published for red sorghum, cereals, and cereal-derived foods using triple quadrupole MS methods [32,33].

As useful additional criterion, the accuracy profile was graphically visualized and checked for each mycotoxin [31]. Nearly all values fall within the acceptance limits (± 20%) and expectedly displayed a decreasing trend with increase in concentration (data not shown). The matrix-matched calibration curves revealed good linearity within the respective spiking ranges with coefficients of determination values (*R*^2^) between 0.9676 and 0.9865 (Table 3). In addition, residuals were randomly distributed around zero (data not shown).

The specificity of the method defined as the ability to distinguish between an analyte and other substances [30] was confirmed for both matrices due to the absence of signal interferences close to the retention times of the mycotoxins.

Another requirement for validated mass spectrometric methods is the confirmation of compound identity by detecting a specified number of product ions. In case of HRMS, the detection of one product ion is sufficient for confirmation purposes [27]. The full MS/data-dependent acquisition (full MS/dd-MS/MS) mode in the present method enabled the detection of a product ion spectra for each mycotoxin, even at low concentration levels. Despite only one product ion being required, we nevertheless decided to identify two product ions for each mycotoxin as common in triple quadrupole MS methods, as this condition is expected to provide more reliable and accurate results. The masses of the precursor ion and the two used product ions are presented in Table S1 for each mycotoxin (Supplementary Material).

The sensitivity of a method is commonly specified by the limit of detection (LOD) and limit of quantification (LOQ), as it is required for food by Commission Regulation (EC) No. 401/2006 [34]. However, specific performance criteria have not been set for feed. Thus, methods for the determination of mycotoxins in feed should be validated according to Commission Decision 2002/657/EC [30].

Hence, CCα and CCβ have to be determined instead of LOD and LOQ [31]. Since the EU has only set guidance values for *Fusarium* mycotoxins in products intended for animal feeding by Commission Recommendation 2006/576/EC [35], CCα and CCβ values were calculated using an approach for substances without a defined maximum limit (cf. Materials and Methods). The CCα values for forage maize and maize silage ranged from 16 to 75 µg/kg and 11 to 88 µg/kg, respectively, whereas the CCβ values varied in a range from 26 to 141 µg/kg and 20 to 125 µg/kg, respectively (Table 3). Compared to the detection limits of previously reported LC-MS/MS methods for the analysis of mycotoxins in maize silage [18,19,36,37], the CCβ values of the present method are mostly in a similar range, indicating a high and comparable sensitivity of the proposed HRMS method (summarized in Table S2, Supplementary Material). Further, the obtained CCβ values for DON and ZEN were far below the guidance values for complementary and complete feeding stuffs (5000 µg/kg DON; 500 µg/kg ZEN) set in Commission Recommendation 2006/576/EC [35], and thus matched the regulatory requirements for the official control.

Overall, the HRMS method fulfilled all the required performance characteristics established in Commission Decision 2002/657/EC [30]. Therefore, HRMS is a sensitive tool for the routine analysis of mycotoxins in complex feed matrices.

### 2.5. Application of the HRMS Method to Real Samples

To evaluate the suitability of the developed HRMS method in real samples, the validated method was applied to a set of naturally contaminated forage maize and maize silage samples collected in Schleswig-Holstein (Northern Germany). A summary of the results is presented in Table 4, and a complete list of results is available as supplementary material (Table S3).

Overall, the incidence of mycotoxins was high in both forage maize and maize silage. At least four mycotoxins were detected in each sample (Table S3). The most frequently found mycotoxins were deoxynivalenol (DON) and zearalenone (ZEN), which were present in all of the analyzed samples with high contents up to 10,972 µg/kg and 1,725 µg/kg, respectively. Notably, the guidance value for ZEN in complementary and complete feeding stuffs (500 µg/kg ZEN) set in Commission Recommendation 2006/576/EC [35] was exceeded in more than half the samples (26 out of 48 samples). The guidance value for DON (5000 µg/kg) was only exceeded in four samples, all of which also exhibited a ZEN value above the guidance value (Table S3).

In addition, samples were contaminated by several DON and ZEN related forms, respectively. The ZEN derivatives α-ZEL and β-ZEL were detected in both types of samples with relatively high incidences and levels (Table 4). The DON related forms 3- and 15-acetyl-DON (3+15-AcDON) were present in all samples that contained DON up to a maximum concentration of 1,832 µg/kg (Table 4). The average concentration of 3+15-AcDON in forage maize (609 µg/kg) was noticeably higher than in maize silage (50 µg/kg). The bacterial DON derivative deepoxy-DON (DOM-1) was neither detected in forage maize nor in maize silage. Considerable amounts of the DON metabolite DON3G were found in all the forage maize samples analyzed. In contrast, DON3G was present in only six out of 27 maize silage samples, and the level never exceeded the decision capability (Table S3). Due to the frequent detection of DON and ZEN-related forms in partially high concentrations in both forage maize and maize silage, *Fusarium* mycotoxin derivatives should receive more attention in further research.

The capability of HRMS to identify novel structurally modified mycotoxins by performing retrospective analysis, i.e., to re-evaluate raw full HRMS data for mycotoxin derivatives without the need to re-measure the sample, is expected to strongly improve research in the field of modified mycotoxins [1]. However, to assess the entire pool of mycotoxins in a food or feed sample, there is the need for non-selective sample preparation procedures. The sample preparation of the present method is based on a generic extraction solvent mixture (acetonitrile/water/acetic acid [79/20/1 (*v*/*v*/*v*)]), which is known to be appropriate for the extraction of a variety of mycotoxins [38], and a simple clean-up step using Bond Elut Mycotoxin^®^ columns. The sorbent is a proprietary silica-based ion exchange material that enables the efficient clean-up of complex extracts without the loss of mycotoxins of interest in a wide polarity range [39]. Given these facts, the present HRMS method allows the retrospective analysis of a variety of chemically diverse mycotoxins, and thus is a powerful platform for the detection of known and unknown mycotoxins in complex feed matrices.

## 3. Conclusions

This study investigated the suitability of Orbitrap^TM^-based HRMS for the confirmatory and quantitative analysis of eight *Fusarium* mycotoxins in highly complex feed matrices. Due to the high amount of co-extracts in complex matrices, the resolving power of the MS method is a critical parameter. In the case of forage maize, a resolving power of 35,000 FWHM was sufficient, whereas a higher resolving power (70,000 FWHM) was found to be required for the fermented matrix maize silage. The developed method based on Orbitrap^TM^ HRMS for the quantification of mycotoxins in forage maize and maize silage was validated according to Commission Decision 2002/657/EC. All the performance characteristics (recovery, precision, CCα, CCβ, compound identification) met the legislation requirements. The reliability of the HRMS method was confirmed by the analysis of naturally contaminated samples collected in northern Germany. At least four mycotoxins were quantified in each sample, highlighting the frequent co-occurrence of mycotoxins in feed and underscoring the importance of regular monitoring of mycotoxin levels.

Overall, Orbitrap^TM^-based HRMS is a robust and reliable instrument for the quantitative analysis of mycotoxins in highly complex feed matrices. It offers the additional advantage of non-target screening and retrospective data mining possibilities, which is a valuable tool due to the lack of analytical standards for a variety of known mycotoxins.

## 4. Materials and Methods

### 4.1. Chemicals

Methanol, water (both LC-MS grade), and acetonitrile (HPLC-grade) were obtained from VWR International (Darmstadt, Germany), ammonium acetate and formic acid (>98%) were supplied by Carl Roth (Karlsruhe, Germany). Corning^TM^ Costar^TM^ Spin-X^TM^ centrifuge tube filters (0.22 µm) were purchased from Fisher Scientific (Schwerte, Germany). Pierce^®^ LTQ Velos ESI positive ion calibration solution and Pierce^®^ ESI negative ion calibration solution were supplied from ThermoFisher Scientific (Rockford, IL, USA). Bond Elut Mycotoxin^®^ cartridges were purchased from Agilent (Santa Clara, CA, USA), mycotoxin standards of deoxynivalenol (DON), 3-acetyl-deoxynivalenol (3-AcDON), 15-acetyl-deoxynivalenol (15-AcDON), deoxynivalenol-3-glucoside (DON3G), deepoxy-deoxynivalenol (DOM-1), and zearalenone (ZEN) were purchased from Romer Labs (Tulln, Austria), while alpha-zearalenol (α-ZEL), beta-zearalenol (β-ZEL), and verrucarol (VER) were supplied by Sigma Aldrich (Saint Louis, MI, USA). DOM-1 and DON3G were obtained in acetonitrile at 50 µg/mL concentration. Stock solutions of DON, 3-AcDON, 15-AcDON, α-ZEL, β-ZEL, ZEN, and VER were prepared in acetonitrile at a concentration of 1 mg/mL. A standard mixture, which was renewed monthly, was prepared in acetonitrile from stock solutions. The standard mixture contained 40 ng/µL DON, 10 ng/µL 3-AcDON, 10 ng/µL 15-AcDON, 40 ng/µL DON3G, 10 ng/µL DOM-1, 20 ng/µL ZEN, 50 ng/µl α-ZEL, and 40 ng/µL β-ZEL. All stock solutions and the standard mixture were stored at −18 °C in the dark and brought to room temperature before use.

### 4.2. Sample Preparation

Initially, 5.00 ± 0.01 g of a homogenized sample were placed in a 50-mL polypropylene centrifuge tube. Each sample was spiked with 20 µL of the internal standard verrucarol (100 µg/mL), vortexed for 30 s, and left to soak for 30 min in the dark. Subsequently, 40 mL of extraction solvent (acetonitrile/water/acetic acid 79/20/1, *v*/*v*/*v*) were added. Then, the samples were extracted for 60 min using a compact shaker (Edmund Bühler, Hechingen, Germany) and centrifuged for 10 min at 3260 g using a Heraeus^TM^ Megafuge^TM^ 8R (ThermoFisher Scientific, Osterrode, Germany). Four milliliters of the sample extract were transferred onto a Bond Elut Mycotoxin^®^ column, mounted on a vacuum manifold, and the sample was eluted. The respective eluate was evaporated to dryness at 40 °C using a SA-VC-300 H vacuum concentrator (H. Saur, Reutlingen, Germany), reconstituted with 300 µL of methanol/water (70/30, *v*/*v*) and vortexed for 1 min. The reconstituted extract was filtered through a centrifuge tube filter.

### 4.3. Samples

Sampling took place in regions in Schleswig-Holstein (Northern Germany) with intensive animal husbandry. Forage maize samples were collected at seven locations directly at harvest in 2017. Samples of approximately 1 kg were taken from chopped material at three different positions in the field. Maize silage samples were collected from nine dairy farms. Approximately 1 kg was taken in triplicate per silo after three months of ensiling 1 m behind the cut surface of the silo using a metal core sampler. The forage maize as well as the maize silage samples were dried (two days at 60 °C) and ground (particle size 1 mm) using an Ultra Centrifugal Mill ZM 200 (Retsch, Haan, Germany). Until mycotoxin analysis, the samples were stored at –18 °C in the dark. The sample preparation was carried out as described above. Each sample (*n* = 48) was analyzed once. If determined concentrations exceeded the range of the matrix-matched calibration curve, the samples as well as the matrix-matched standards were diluted with reconstitution solvent (methanol/water [70/30, *v*/*v*]) and re-analyzed. For a correct mycotoxin identification in the samples, the following criteria based on the recent document SANTE/12089/2016 [27] had to be fulfilled: (i) precursor ions had to be monitored with a mass accuracy ≤5 ppm, (ii) two product ions had to be detected, (iii) the ion ratio had to be within ± 30% to that obtained for the calibration standards average, and (iv) the retention time had to match the time window from that of the average of the calibration standards with a tolerance of ± 0.1 min.

### 4.4. LC-HRMS

LC-HRMS analysis was performed using a Dionex UltiMate^®^ 3000 coupled to a Q-Exactive^®^ mass spectrometer (ThermoFisher Scientific, Bremen, Germany). The chromatographic separation of *Fusarium* mycotoxins was achieved using an XBridge^TM^ C_18_ column, 100 × 2.1 mm i.d., 3.5 µm particle size, equipped with an XBridge^TM^ C_18_ 5 × 2.1 mm i.d. guard column (all from Waters, Milford, MA, USA). Water (A) and methanol (B), both containing 0.1% formic acid and 5 mM ammonium acetate, were used as mobiles phases. The flow rate of the mobile phase was set to 0.3 mL/min. The gradient profile was as follows: 0.0 min–3% B, 1.0 min–3% B, 9.0 min–80% B, 9.5 min–97% B, 10.5 min–97% B, 11.0 min–3% B, 14.0 min–3% B. Five microliters of standard solution, forage maize, or silage maize extract were injected in the system. The column and autosampler were kept at 25 °C and 10 °C, respectively.

During ionization efficiency experiments, the heated electrospray ionization (HESI) as well as the atmospheric pressure chemical ionization (APCI) interface were used. When using the HESI interface, the following instrumental settings were applied: sheath, auxiliary, and sweep gas flow rates, 32, 7, and 0 arbitrary units, respectively; spray voltage, 3.3 kV; heater temperature, 220 °C; capillary temperature, 300 °C; S-lens level, 60 arbitrary units. The APCI interface operated with the following instrumental settings: sheath, auxiliary, and sweep gas flow rates, 35, 10, and 0, arbitrary units, respectively; discharge voltage, 5 kV; S-lens level, 60 arbitrary units; capillary temperature, 250 °C; and vaporizer temperature, 250 °C.

The Q-Exactive^®^ mass spectrometer operated in full MS/data-dependent MS/MS mode (full MS/dd-MS/MS). The full MS mode acquired data for the quantification, while the dd-MS/MS mode provided diagnostic product ions that were used for the confirmation of the mycotoxin identity. The following settings were used in full MS mode: resolution 70,000 FWHM (defined for m/z 200; 3 Hz), scan range 200–600 m/z, automatic gain control (AGC) target 1e6, maximum inject time (IT) 100 ms. The dd-MS/MS mode utilized the following settings: resolution 70,000 FWHM (defined for m/z 200; 3 Hz), scan range 200–600 m/z, AGC target 1e5, IT 200 ms, isolation window 1 m/z, and dynamic exclusion 5 s. Fragmentation was achieved using a stepped collision energy setting of 20 eV and 60 eV. Mycotoxin signals were extracted from the raw data using a mass extraction window of ± 5 ppm. During resolving power experiments, a wider mass extraction window was applied (± 20 ppm).

A mass calibration of the mass spectrometer was regularly performed in three-day intervals and before each measurement sequence using calibration solution and Thermo TunePlus^®^ 2.8 software (ThermoFisher Scientific). Xcalibur 4.0^®^ software (ThermoFisher Scientific) was used for data acquisition and TraceFinder^®^ 4.1 software (ThermoFisher Scientific) was used for data processing.

### 4.5. Method Validation

The LC-HRMS method was validated for forage maize and maize silage according to the Commission Decision 2002/657/EC [30] guidelines in terms of linearity, apparent recovery (R_A_), intra-day precision (repeatability; RSD_r_), inter-day precision (intermediate precision; RSD_R_), specificity, measurement uncertainty (U), decision limit (CCα), and detection capability (CCβ) by spiking blank samples.

Five blank samples of each matrix were spiked with a mycotoxin mixture solution at five different concentration levels, namely 0.5, 0.75, 1, 1.5, and 2 times the cutoff level. This procedure was carried out in triplicate on three consecutive days. Guidance values for *Fusarium* mycotoxins in products intended for animal feed were established by Commission Recommendation 2006/576/EC [35], but no minimum required performance limits (MRPLs) were defined for mycotoxins in feed. For this reason, the ‘cutoff level’ approach introduced by Monbaliu et al. [40] was adopted. A cutoff level was established for each mycotoxin near the quantification limit.

The samples used as blanks for the spiking experiments were collected in northern Germany in 2016. They exhibited a mycotoxin content lower than one-fourth of the cutoff level. A matrix blank was included in each batch of samples, and the peak area of each mycotoxin in the test sample was corrected by subtracting the respective mycotoxin peak area in the matrix blank sample. Matrix-matched calibration curves were obtained by plotting the relative peak area (peak area mycotoxin/peak area internal standard [VER]) versus the spiked concentration. Theoretically, the use of isotope-labeled internal standards is preferred, since they share the same physicochemical properties as the target mycotoxins. However, their use was not feasible due to the high costs. Therefore, the structurally related mycotoxin verrucarol was added in this study as an internal standard to correct for losses during extraction and clean-up.

Linearity was determined for each matrix and mycotoxin by fitting a linear model and confirmed by residual plot calculation. The coefficient of determination (*R*^2^) was calculated using the means of the least square approach.

To check the specificity of the method, 20 independent blank samples were analyzed for each matrix. The apparent recovery (*R*_A_) was determined as the ratio of the concentration value calculated with the matrix-matched calibration curve divided by the spiked concentration value. The precision was evaluated by calculating the relative standard deviations (RSD). For the intra-day precision (RSD_r_) of the method, five samples with five different concentration levels were analyzed in triplicate on the same day. For the inter-day precision (RSD_R_), the same procedure was repeated on three consecutive days.

The decision limit (CCα) and detection capability (CCβ) were estimated using the matrix-matched calibration curves. CCα was determined as the “corresponding concentration at the y-intercept plus 2.33× the standard deviation of RSD_R_” [30]. CCβ was calculated as the “concentration at the decision limit plus 1.64× the standard deviation of RSD_R_” [30].

The validation data were also used to develop an accuracy profile for each mycotoxin. The bias acceptable limit λ has been fixed to ± 20% with a beta error of 0.90. Additionally, the validation parameters of intra-day precision, inter-day precision, and bias estimates were used to calculate the expanded measurement uncertainty (*U*) according to the recommendations of the ISO/TS 21748:2017 guide [41]. The expanded measurement uncertainty (*U*) was calculated by multiplying the combined standard uncertainty *(u_c_*) by a coverage factor (*k*). To obtain a level of confidence of approximately 95%, a coverage factor of two was applied. The combined standard uncertainty (*u_c_*) included the variance of the inter-day precision (*s^2^_R_*) and the uncertainty associated with the bias (*u_bias_^2^*). The uncertainty associated with the bias was calculated using the variance of the intra-day precision (*s^2^_r_*), the variance of the inter-day precision (*s^2^_R_*), the number of replicates (*n*), and the number of different conditions (*p*).

The Equations used were as follows:

(1) $$U=k\;x\;u_{c}=k\;x\;\sqrt{s_{R}{}^{2}+u_{bias}^{2}}$$

(2) $$u_{bias}=\;\sqrt{\frac{s_{R}^{2}-\left(1-1/n\right)s_{r}^{2}}{p}}$$

### 4.6. Data Analysis

The calculations were executed using Microsoft Excel^®^ 2016 (Microsoft Corporation, Redmond, WA, USA) and the statistical software R^®^ 3.3.3 (R Foundation for Statistical Computing, Vienna, Austria). The mass accuracy, expressed in parts per million, was calculated by dividing the difference between measured mass and theoretical mass by the theoretical mass.

## Figures and Tables

**Table 1:** Number of mycotoxins (including the internal standard verrucarol) detected with stated mass accuracy (in ppm) for each resolving power and concentration level combination in forage maize and maize silage.

Concentration Level (µg/kg)	Resolving Power															
	17.500 FWHM ^a^				35.000 FWHM ^a^				70.000 FWHM ^a^				140.000 FWHM ^a^			
	<2 ppm	2–5 ppm	5–10 ppm	>10 ppm	<2 ppm	2–5 ppm	5–10 ppm	>10 ppm	<2 ppm	2–5 ppm	5–10 ppm	>10 ppm	<2 ppm	2–5 ppm	5–10 ppm	>10 ppm
Forage maize																
100	3	2	2	2	3	6	0	0	9	0	0	0	9	0	0	0
200	2	3	3	1	6	3	0	0	9	0	0	0	9	0	0	0
300	6	1	2	0	9	0	0	0	9	0	0	0	9	0	0	0
Maize silage																
100	1	2	3	3	3	0	5	1	9	0	0	0	9	0	0	0
200	1	5	0	3	5	2	1	0	9	0	0	0	9	0	0	0
300	3	3	2	1	8	1	0	0	9	0	0	0	9	0	0	0

^a^ full width at half maximum (FWHM) at *m/z* 200; *n* = nine mycotoxins (including internal standard).

**Table 2:** Validation results of the HRMS method at the lowest, medium, and highest concentration level in forage maize and maize silage; apparent recovery (R_A_), intra-day precision (RSD_r_), inter-day precision (RSD_R_), and measurement uncertainty (U).

Mycotoxin	Spiked Concentration (µg/kg)	Forage Maize				Maize Silage			
		R_A_ (%)	RSD_r_ (%)	RSD_R_ (%)	U (%)	R_A_ (%)	RSD_r_ (%)	RSD_R_ (%)	U (%)
DON	200	98	11	11	24	98	13	2	26
	400 *	106	7	5	15	101	6	7	20
	800	102	6	5	11	101	5	2	10
DON3G	200	103	15	10	36	103	16	8	42
	400 *	106	11	9	29	94	11	2	24
	800	104	9	11	24	96	14	5	22
DOM-1	50	96	10	3	23	99	12	3	15
	100 *	108	7	4	17	95	5	4	13
	200	99	4	2	8	101	3	2	7
3+15-AcDON	50	101	9	8	13	100	6	3	13
	100 *	104	6	5	16	98	5	3	12
	200	97	5	4	13	101	3	2	7
β-ZEL	200	105	11	8	22	96	9	6	21
	400 *	106	5	6	13	96	3	3	8
	800	100	6	3	12	99	2	3	7
α-ZEL	250	95	8	12	31	104	16	7	41
	500 *	104	9	7	22	103	5	7	18
	1000	98	2	3	8	102	4	2	8
ZEN	100	97	11	8	25	100	7	6	18
	200 *	105	8	6	20	98	6	3	14
	400	99	3	3	8	100	5	2	10

* cutoff level; DON = deoxynivalenol; DON3G = deoxynivalenol-3-glucoside; DOM-1 = deepoxy-deoxynivalenol; 3-AcDON = 3-acetyl-deoxynivalenol; 15-AcDON = 15-acetyl-deoxynivalenol; β-ZEL = β-zearalenol; α-ZEL = α-zearalenol; ZEN = zearalenone.

**Table 3:** Results for coefficients of determination (*R^2^*), decision limits (CCα), and detection capabilities (CCβ) obtained for the analyzed mycotoxins in forage maize and maize silage.

Mycotoxin	Range (µg/kg)	Forage Maize			Maize Silage		
		*R^2^* (mean)	CCα (µg/kg)	CCβ (µg/kg)	*R^2^* (mean)	CCα (µg/kg)	CCβ (µg/kg)
DON	200–800	0.9736	75	141	0.9865	47	82
DON3G	200–800	0.9768	17	29	0.9845	63	94
DOM-1	50–200	0.9809	16	31	0.9790	15	31
3+15-AcDON	50–200	0.9764	18	28	0.9839	11	20
β-ZEL	200–800	0.9676	73	108	0.9864	46	90
α-ZEL	250–1000	0.9814	16	26	0.9684	88	125
ZEN	100–400	0.9765	36	60	0.9740	40	61

DON = deoxynivalenol; DON3G = deoxynivalenol-3-glucoside; DOM-1 = deepoxy-deoxynivalenol; 3-AcDON = 3-acetyl-deoxynivalenol; 15-AcDON = 15-acetyl-deoxynivalenol; β-ZEL = β-zearalenol; α-ZEL = α-zearalenol; ZEN = zearalenone.

**Table 4:** Incidence (%) and concentrations of *Fusarium* mycotoxins (µg/kg) detected in forage maize and maize silage samples (*n* = 48) collected in Schleswig-Holstein (Northern Germany).

	DON	DON3G	DOM-1	3+15-AcDON	β-ZEL	α-ZEL	ZEN
**Forage maize (*n* = 21)**							
Incidence (%)	100	100	0	100	81	95	100
Mean (µg/kg) ^a^	2794	574	n.a.	609	149	110	991
Min (µg/kg) ^a^	466	119	n.d.	29	135	28	66
Max (µg/kg) ^a^	10972	1240	n.d.	1832	163	423	1725
CCβ	141	29	31	28	60	26	108
**Maize Silage (*n* = 27)**							
Incidence (%)	100	22	0	100	85	89	97
Mean (µg/kg) ^a^	2051	n.a.	n.a.	50	-	221	527
Min (µg/kg) ^a^	265	<CCβ	n.d.	21	<CCβ	178	63
Max (µg/kg) ^a^	5401	<CCβ	n.d.	149	<CCβ	339	1596
CCβ	82	94	31	20	61	125	90

^a^ Mean, minimum, and maximum mycotoxin concentration of the positive (>CCβ) samples; n.d. not detected; n.a. not applicable; CCβ = detection capability; DON = deoxynivalenol; DON3G = deoxynivalenol-3-glucoside; DOM-1 = deepoxy-deoxynivalenol; 3-AcDON = 3-acetyl-deoxynivalenol; 15-AcDON = 15-acetyl-deoxynivalenol; β-ZEL = β-zearalenol; α-ZEL = α-zearalenol; ZEN = zearalenone.

## Supplementary Materials

The following are available online at https://www.mdpi.com/2072-6651/11/9/531/s1, Table S1: LC-HRMS parameters for the detection of *Fusarium* mycotoxins, including the retention time, analyte formula, molecular ion, precursor ion, and product ions. Table S2: Detection limits (µg/kg) of *Fusarium* mycotoxins in maize silage. Comparison of published LC-MS/MS methodologies and the proposed LC-HRMS method. Table S3: Concentrations (µg/kg ± U) of the detected mycotoxins in forage maize and maize silage samples collected in Northern Germany (*n* = 48).
