# Supplementary material for: Evaluation of High-Resolution Mass Spectrometry for the Quantitative Analysis of Mycotoxins in Complex Feed Matrices
Source: Toxins (Basel). 2019 Sep 12;11(9):531. doi: 10.3390/toxins11090531 (PMC6783880; doi:10.3390/toxins11090531)
Supplement: Supplementary file 1 [file toxins-11-00531-s001.zip › toxins-590446-supplementary publish/toxins-590446-supplementary publish.docx]

Supplementary Materials:Evaluation of High-Resolution Mass Spectrometry for the Quantitative Analysis of Mycotoxins in Complex Feed Matrices’

Tolke Jensen, Marthe de Boevre, Nils Preußke, Sarah de Saeger, Tim Birr, Joseph-Alexander Verreet and Frank D. Sönnichsen

**Table S1.** LC-HRMS parameters for the detection of *Fusarium* mycotoxins, including the retention time, analyte formula, molecular ion, precursor ion and product ions.

| **Mycotoxin** | **Retention time (min)** | **Formula** | **Molecular ion** | **Precursor ion *(m/z)*** | **Product ions *(m/z)*** |
| --- | --- | --- | --- | --- | --- |
| DON | 5.51 | C_15_H_20_O_6_ | [M+H]^+^ | 297.1333 | 203.1066, 231.1017 |
| DON3G | 5.64 | C_21_H_30_O_11_ | [M+NH_4_]^+^ | 476.2126 | 249.1121, 279.1227 |
| DOM-1 | 6.51 | C_15_H_20_O_5_ | [M+H]^+^ | 281.1384 | 109.0651, 215.1065 |
| VER* | 6.93 | C_15_H_22_O_4_ | [M+H]^+^ | 267.1591 | 219.1379, 213.1273 |
| 3-AcDON | 7.38 | C_17_H_22_O_7_ | [M+H]^+^ | 339.1144 | 321.1331, 189.0911 |
| 15-AcDON | 7.38 | C_17_H_22_O_7_ | [M+H]^+^ | 339.1144 | 137.0598, 189.0911 |
| β-ZEL | 9.95 | C_18_H_24_O_5_ | [M+H]^+^ | 321.1697 | 285.1484, 303.1592 |
| α-ZEL | 10.45 | C_18_H_24_O_5_ | [M+H]^+^ | 321.1697 | 285.1484, 267.1381 |
| ZEN | 10.60 | C_18_H_22_O_5_ | [M+H]^+^ | 319.1540 | 283.1330, 187.0755 |

^*^ used as internal standard; DON = deoxynivalenol; DON3G = deoxynivalenol-3-glucoside; DOM-1 = deepoxy-deoxynivalenol; 3-AcDON = 3-acetyl-deoxynivalenol; 15-AcDON = 15-acetyl-deoxynivalenol; β-ZEL = β-zearalenol; α-ZEL = α-zearalenol; ZEN = zearalenone

**Table S2.** Detection limits (µg/kg) of *Fusarium* mycotoxins in maize silage. Comparison of published LC-MS/MS methodologies and the proposed LC-HRMS method.

| **Ref.** | **Detector** | **Basis of calculation** | **Detection limit (µg/kg)** | | | | | | |
| --- | --- | --- | --- | --- | --- | --- | --- | --- | --- |
|  |  |  | **DON** | **DON3G** | **DOM-1** | **3+15-AcDON** | **β-ZEL** | **α-ZEL** | **ZEN** |
| [1] | LC-MS/MS | LOQ | 739 | -^a^ | -^a^ | -^a^ | -^a^ | -^a^ | 9 |
| [2] | LC-MS/MS | LOQ | 99 | -^a^ | -^a^ | -^a^ | 64 | 64 | 23 |
| [3] | LC-MS/MS | LOQ | 100 | 50 | -^a^ | 100 | 2.5 | 2.5 | 1 |
| [4] | LC-MS/MS | CCβ | 1072 | -^a^ | -^a^ | 1109 | 237 | 288 | 135 |
| Current article | LC-HRMS | CCβ | 82 | 94 | 31 | 20 | 90 | 125 | 61 |

^a^ not included in study; LOQ = limit of quantification; CCβ = detection capability; DON = deoxynivalenol; DON3G = deoxynivalenol-3-glucoside; DOM-1 = deepoxy-deoxynivalenol; 3-AcDON = 3-acetyl-deoxynivalenol; 15-AcDON = 15-acetyl-deoxynivalenol; β-ZEL = β-zearalenol; α-ZEL = α-zearalenol; ZEN = zearalenone

**Table S3.** Concentrations (µg/kg ± U) of the detected mycotoxins in forage maize and maize silage samples collected in Northern Germany (n = 48).

| **No.** | **Sample type** | **DON** | **DON3G** | **DOM-1** | **3+15-AcDON** | **β-ZEL** | **α-ZEL** | **ZEN** |
| --- | --- | --- | --- | --- | --- | --- | --- | --- |
| 1 | forage maize | 2888 ± 578 | 651 ± 156 | n.d. | 581 ± 93 | < CCβ | 30 ± 9 | 1638 ± 229 |
| 2 | forage maize | 2154 ± 323 | 473 ± 137 | n.d. | 305 ± 40 | 135 ± 30 | < CCβ | 308 ± 43 |
| 3 | forage maize | 1027 ± 247 | 261 ± 94 | n.d. | 168 ± 29 | < CCβ | < CCβ | 201 ± 40 |
| 4 | forage maize | 466 ± 70 | 119 ± 42 | n.d. | 29 ± 4 | n.d. | < CCβ | < CCβ |
| 5 | forage maize | 653 ± 131 | 121 ± 44 | n.d. | 59 ± 8 | < CCβ | n.d. | 66 ± 17 |
| 6 | forage maize | 1087 ± 261 | 184 ± 66 | n.d. | 259 ± 34 | < CCβ | < CCβ | 462 ± 40 |
| 7 | forage maize | 2141 ± 321 | 449 ± 130 | n.d. | 460 ± 74 | < CCβ | 28 ± 9 | 1414 ± 198 |
| 8 | forage maize | 3488 ± 384 | 1165 ± 280 | n.d. | 602 ± 96 | < CCβ | < CCβ | 1236 ± 173 |
| 9 | forage maize | 2528 ± 379 | 694 ± 167 | n.d. | 398 ± 88 | < CCβ | 90 ± 28 | 1644 ± 230 |
| 10 | forage maize | 794 ± 87 | 271 ± 98 | n.d. | 91 ± 15 | n.d. | < CCβ | 603 ± 151 |
| 11 | forage maize | 800 ± 88 | 198 ± 71 | n.d. | 196 ± 26 | < CCβ | < CCβ | 638 ± 159 |
| 12 | forage maize | 1261 ± 302 | 342 ± 99 | n.d. | 230 ± 30 | < CCβ | 41 ± 13 | 1299 ± 182 |
| 13 | forage maize | 10972 ± 1207 | 1167 ± 280 | n.d. | 1799 ± 234 | 163 ± 36 | 423 ± 68 | 1569 ± 220 |
| 14 | forage maize | 1034 ± 248 | 149 ± 54 | n.d. | 237 ± 31 | < CCβ | < CCβ | 391 ± 31 |
| 15 | forage maize | 4949 ± 544 | 917 ± 220 | n.d. | 1144 ± 149 | n.d. | 259 ± 80 | 810 ± 203 |
| 16 | forage maize | 3268 ± 654 | 841 ± 202 | n.d. | 969 ± 126 | < CCβ | 83 ± 26 | 1334 ± 187 |
| 17 | forage maize | 1134 ± 272 | 293 ± 105 | n.d. | 303 ± 39 | < CCβ | < CCβ | 265 ± 37 |
| 18 | forage maize | 5269 ± 580 | 894 ± 214 | n.d. | 1173 ± 152 | < CCβ | 88 ± 28 | 1725 ± 242 |
| 19 | forage maize | 1668 ± 334 | 584 ± 169 | n.d. | 781 ± 133 | n.d. | 35 ± 8 | 937 ± 187 |
| 20 | forage maize | 3382 ± 676 | 1044 ± 251 | n.d. | 1165 ± 151 | < CCβ | 83 ± 26 | 1351 ± 189 |
| 21 | forage maize | 7704 ± 847 | 1240 ± 298 | n.d. | 1832 ± 238 | < CCβ | 56 ± 17 | 925 ± 185 |
| 22 | maize silage | 4035 ± 404 | n.d. | n.d. | 30 ± 4 | < CCβ | < CCβ | 1123 ± 157 |
| 23 | maize silage | 1893 ± 379 | < CCβ | n.d. | 30 ± 4 | < CCβ | 187 ± 77 | 446 ± 45 |
| 24 | maize silage | 2764 ± 359 | n.d. | n.d. | 24 ± 3 | n.d. | 186 ± 76 | 564 ± 102 |
| 25 | maize silage | 1312 ± 341 | n.d. | n.d. | < CCβ | < CCβ | n.d. | 63 ± 11 |
| 26 | maize silage | 595 ± 77 | n.d. | n.d. | < CCβ | < CCβ | < CCβ | 392 ± 39 |
| 27 | maize silage | 1021 ± 265 | < CCβ | n.d. | < CCβ | < CCβ | n.d. | 75 ± 14 |
| 28 | maize silage | 1130 ± 294 | < CCβ | n.d. | 31 ± 4 | < CCβ | < CCβ | 147 ± 31 |
| 29 | maize silage | 2705 ± 541 | n.d. | n.d. | 39 ± 5 | < CCβ | < CCβ | 267 ± 59 |
| 30 | maize silage | 2737 ± 547 | n.d. | n.d. | < CCβ | < CCβ | < CCβ | 1375 ± 303 |
| 31 | maize silage | < CCβ | n.d. | n.d. | < CCβ | < CCβ | < CCβ | < CCβ |
| 32 | maize silage | 407 ± 81 | n.d. | n.d. | 33 ± 4 | < CCβ | < CCβ | 417 ± 42 |
| 33 | maize silage | 312 ± 56 | n.d. | n.d. | < CCβ | < CCβ | < CCβ | 111 ± 20 |
| 34 | maize silage | 2093 ± 419 | n.d. | n.d. | < CCβ | < CCβ | < CCβ | 555 ± 100 |
| 35 | maize silage | 2306 ± 461 | < CCβ | n.d. | 149 ± 21 | < CCβ | 199 ± 82 | 667 ± 140 |
| 36 | maize silage | 2111 ± 422 | n.d. | n.d. | < CCβ | < CCβ | < CCβ | 537 ± 97 |
| 37 | maize silage | 2156 ± 431 | < CCβ | n.d. | 33 ± 4 | < CCβ | 181 ± 74 | 893 ± 125 |
| 38 | maize silage | 5401 ± 540 | < CCβ | n.d. | 45 ± 6 | n.d. | 275 ± 113 | 956 ± 134 |
| 39 | maize silage | 4675 ± 468 | n.d. | n.d. | 32 ± 4 | < CCβ | 339 ± 125 | 1596 ± 351 |
| 40 | maize silage | 2356 ± 306 | n.d. | n.d. | 21 ± 3 | n.d. | < CCβ | 426 ± 43 |
| 41 | maize silage | 694 ± 90 | n.d. | n.d. | < CCβ | < CCβ | < CCβ | 184 ± 26 |
| 42 | maize silage | 5129 ± 513 | n.d. | n.d. | < CCβ | < CCβ | 178 ± 73 | 852 ± 179 |
| 43 | maize silage | 1044 ± 271 | n.d. | n.d. | 82 ± 14 | < CCβ | < CCβ | 77 ± 14 |
| 44 | maize silage | 409 ± 82 | n.d. | n.d. | < CCβ | < CCβ | < CCβ | 67 ± 12 |
| 45 | maize silage | 3177 ± 413 | n.d. | n.d. | < CCβ | < CCβ | n.d. | 704 ± 148 |
| 46 | maize silage | 2247 ± 449 | n.d. | n.d. | 103 ± 12 | n.d. | < CCβ | 408 ± 41 |
| 47 | maize silage | 349 ± 63 | n.d. | n.d. | < CCβ | < CCβ | < CCβ | 271 ± 60 |
| 48 | maize silage | 265 ± 48 | n.d. | n.d. | < CCβ | < CCβ | < CCβ | 519 ± 93 |

n.d.= not detected; CCβ= detection capability; DON = deoxynivalenol; DON3G = deoxynivalenol-3-glucoside; DOM-1 = deepoxy-deoxynivalenol; 3-AcDON = 3-acetyl-deoxynivalenol; 15-AcDON = 15-acetyl-deoxynivalenol; β-ZEL = β-zearalenol; α-ZEL = α-zearalenol; ZEN = zearalenone

1. Rasmussen, R.R.; Storm, I.M.L.D.; Rasmussen, P.H.; Smedsgaard, J.; Nielsen, K.F. Multi-mycotoxin analysis of maize silage by LC-MS/MS. *Anal. Bioanal. Chem.* **2010**, *397*, 765–776.
2. Van Pamel, E.; Verbeken, A.; Vlaemynck, G.; de Boever, J.; Daeseleire, E. Ultrahigh-performance liquid chromatographic-tandem mass spectrometric multimycotoxin method for quantitating 26 mycotoxins in maize silage. *J. Agric. Food Chem.* **2011**, *59*, 9747–9755.
3. Dzuman, Z.; Zachariasova, M.; Lacina, O.; Veprikova, Z.; Slavikova, P.; Hajslova, J. A rugged high-throughput analytical approach for the determination and quantification of multiple mycotoxins in complex feed matrices. *Talanta* **2014**, *121*, 263–272.
4. Dagnac, T.; Latorre, A.; Fernández Lorenzo, B.; Llompart, M. Validation and application of a liquid chromatography-tandem mass spectrometry based method for the assessment of the co-occurrence of mycotoxins in maize silages from dairy farms in NW Spain. *Food Addit. Contam. Part A* **2016**, *33*, 1850–1863.
