# Supplementary material for: Evaluation of High-Resolution Mass Spectrometry for the Quantitative Analysis of Mycotoxins in Complex Feed Matrices
Source: Toxins (Basel). 2019 Sep 12;11(9):531. doi: 10.3390/toxins11090531 (PMC6783880; doi:10.3390/toxins11090531)
Supplement: Supplementary file 1 [file toxins-11-00531-s001.zip › toxins-590446-supplementary publish/toxins-590446-supplementary publish.pdf]

Supplementary Materials:Evaluation of High-Resolution Mass Spectrometry for the Quantitative Analysis of Mycotoxins in Complex Feed Matrices’

Tolke Jensen, Marthe de Boevre, Nils Preußke, Sarah de Saeger, Tim Birr, Joseph-Alexander Verreet and Frank D. Sönnichsen

Table S1. LC-HRMS parameters for the detection of *Fusarium* mycotoxins, including the retention time, analyte formula, molecular ion, precursor ion and product ions.

Mycotoxin	Retention time (min)	Formula	Molecular ion	Precursor ion (*m/z*)	Product ions (*m/z*)
DON	5.51	$C_{15}H_{20}O_6$	$[M+H]^+$	297.1333	203.1066, 231.1017
DON3G	5.64	$C_{21}H_{30}O_{11}$	$[M+NH_4]^+$	476.2126	249.1121, 279.1227
DOM-1	6.51	$C_{15}H_{20}O_5$	$[M+H]^+$	281.1384	109.0651, 215.1065
VER*	6.93	$C_{15}H_{22}O_4$	$[M+H]^+$	267.1591	219.1379, 213.1273
3-AcDON	7.38	$C_{17}H_{22}O_7$	$[M+H]^+$	339.1144	321.1331, 189.0911
15-AcDON	7.38	$C_{17}H_{22}O_7$	$[M+H]^+$	339.1144	137.0598, 189.0911
β-ZEL	9.95	$C_{18}H_{24}O_5$	$[M+H]^+$	321.1697	285.1484, 303.1592
α-ZEL	10.45	$C_{18}H_{24}O_5$	$[M+H]^+$	321.1697	285.1484, 267.1381
ZEN	10.60	$C_{18}H_{22}O_5$	$[M+H]^+$	319.1540	283.1330, 187.0755

[*] used as internal standard; DON = deoxynivalenol; DON3G = deoxynivalenol-3-glucoside; DOM-1 = deepoxy-deoxynivalenol; 3-AcDON = 3-acetyl-deoxynivalenol; 15-AcDON = 15-acetyl-deoxynivalenol; β-ZEL = β-zearalenol; α-ZEL = α-zearalenol; ZEN = zearalenone

Table S2. Detection limits (µg/kg) of *Fusarium* mycotoxins in maize silage. Comparison of published LC-MS/MS methodologies and the proposed LC-HRMS method.

Ref.	Detector	Basis of calculation	Detection limit (µg/kg)						
			DON	DON3G	DOM-1	3+15-AcDON	β-ZEL	α-ZEL	ZEN
[1]	LC-MS/MS	LOQ	739	-[a]	-[a]	-[a]	-[a]	-[a]	9
[2]	LC-MS/MS	LOQ	99	-[a]	-[a]	-[a]	64	64	23
[3]	LC-MS/MS	LOQ	100	50	-[a]	100	2.5	2.5	1
[4]	LC-MS/MS	CCβ	1072	-[a]	-[a]	1109	237	288	135
Current article	LC-HRMS	CCβ	82	94	31	20	90	125	61

[a] not included in study; LOQ = limit of quantification; CCβ = detection capability; DON = deoxynivalenol; DON3G = deoxynivalenol-3-glucoside; DOM-1 = deepoxy-deoxynivalenol; 3-AcDON = 3-acetyl-deoxynivalenol; 15-AcDON = 15-acetyl-deoxynivalenol; β-ZEL = β-zearalenol; α-ZEL = α-zearalenol; ZEN = zearalenone

Table S3. Concentrations (µg/kg ± U) of the detected mycotoxins in forage maize and maize silage samples collected in Northern Germany (n = 48).

No.	Sample type	DON	DON3G	DOM-1	3+15-AcDON	β-ZEL	α-ZEL	ZEN
1	forage maize	2888 ± 578	651 ± 156	n.d.	581 ± 93	< CCβ	30 ± 9	1638 ± 229
2	forage maize	2154 ± 323	473 ± 137	n.d.	305 ± 40	135 ± 30	< CCβ	308 ± 43
3	forage maize	1027 ± 247	261 ± 94	n.d.	168 ± 29	< CCβ	< CCβ	201 ± 40
4	forage maize	466 ± 70	119 ± 42	n.d.	29 ± 4	n.d.	< CCβ	< CCβ
5	forage maize	653 ± 131	121 ± 44	n.d.	59 ± 8	< CCβ	n.d.	66 ± 17
6	forage maize	1087 ± 261	184 ± 66	n.d.	259 ± 34	< CCβ	< CCβ	462 ± 40
7	forage maize	2141 ± 321	449 ± 130	n.d.	460 ± 74	< CCβ	28 ± 9	1414 ± 198
8	forage maize	3488 ± 384	1165 ± 280	n.d.	602 ± 96	< CCβ	< CCβ	1236 ± 173
9	forage maize	2528 ± 379	694 ± 167	n.d.	398 ± 88	< CCβ	90 ± 28	1644 ± 230
10	forage maize	794 ± 87	271 ± 98	n.d.	91 ± 15	n.d.	< CCβ	603 ± 151
11	forage maize	800 ± 88	198 ± 71	n.d.	196 ± 26	< CCβ	< CCβ	638 ± 159
12	forage maize	1261 ± 302	342 ± 99	n.d.	230 ± 30	< CCβ	41 ± 13	1299 ± 182
13	forage maize	10972 ± 1207	1167 ± 280	n.d.	1799 ± 234	163 ± 36	423 ± 68	1569 ± 220
14	forage maize	1034 ± 248	149 ± 54	n.d.	237 ± 31	< CCβ	< CCβ	391 ± 31
15	forage maize	4949 ± 544	917 ± 220	n.d.	1144 ± 149	n.d.	259 ± 80	810 ± 203
16	forage maize	3268 ± 654	841 ± 202	n.d.	969 ± 126	< CCβ	83 ± 26	1334 ± 187
17	forage maize	1134 ± 272	293 ± 105	n.d.	303 ± 39	< CCβ	< CCβ	265 ± 37
18	forage maize	5269 ± 580	894 ± 214	n.d.	1173 ± 152	< CCβ	88 ± 28	1725 ± 242
19	forage maize	1668 ± 334	584 ± 169	n.d.	781 ± 133	n.d.	35 ± 8	937 ± 187
20	forage maize	3382 ± 676	1044 ± 251	n.d.	1165 ± 151	< CCβ	83 ± 26	1351 ± 189
21	forage maize	7704 ± 847	1240 ± 298	n.d.	1832 ± 238	< CCβ	56 ± 17	925 ± 185
22	maize silage	4035 ± 404	n.d.	n.d.	30 ± 4	< CCβ	< CCβ	1123 ± 157
23	maize silage	1893 ± 379	< CCβ	n.d.	30 ± 4	< CCβ	187 ± 77	446 ± 45
24	maize silage	2764 ± 359	n.d.	n.d.	24 ± 3	n.d.	186 ± 76	564 ± 102
25	maize silage	1312 ± 341	n.d.	n.d.	< CCβ	< CCβ	n.d.	63 ± 11
26	maize silage	595 ± 77	n.d.	n.d.	< CCβ	< CCβ	< CCβ	392 ± 39
27	maize silage	1021 ± 265	< CCβ	n.d.	< CCβ	< CCβ	n.d.	75 ± 14
28	maize silage	1130 ± 294	< CCβ	n.d.	31 ± 4	< CCβ	< CCβ	147 ± 31
29	maize silage	2705 ± 541	n.d.	n.d.	39 ± 5	< CCβ	< CCβ	267 ± 59
30	maize silage	2737 ± 547	n.d.	n.d.	< CCβ	< CCβ	< CCβ	1375 ± 303
31	maize silage	< CCβ	n.d.	n.d.	< CCβ	< CCβ	< CCβ	< CCβ
32	maize silage	407 ± 81	n.d.	n.d.	33 ± 4	< CCβ	< CCβ	417 ± 42
33	maize silage	312 ± 56	n.d.	n.d.	< CCβ	< CCβ	< CCβ	111 ± 20
34	maize silage	2093 ± 419	n.d.	n.d.	< CCβ	< CCβ	< CCβ	555 ± 100
35	maize silage	2306 ± 461	< CCβ	n.d.	149 ± 21	< CCβ	199 ± 82	667 ± 140
36	maize silage	2111 ± 422	n.d.	n.d.	< CCβ	< CCβ	< CCβ	537 ± 97
37	maize silage	2156 ± 431	< CCβ	n.d.	33 ± 4	< CCβ	181 ± 74	893 ± 125
38	maize silage	5401 ± 540	< CCβ	n.d.	45 ± 6	n.d.	275 ± 113	956 ± 134
39	maize silage	4675 ± 468	n.d.	n.d.	32 ± 4	< CCβ	339 ± 125	1596 ± 351
40	maize silage	2356 ± 306	n.d.	n.d.	21 ± 3	n.d.	< CCβ	426 ± 43
41	maize silage	694 ± 90	n.d.	n.d.	< CCβ	< CCβ	< CCβ	184 ± 26
42	maize silage	5129 ± 513	n.d.	n.d.	< CCβ	< CCβ	178 ± 73	852 ± 179
43	maize silage	1044 ± 271	n.d.	n.d.	82 ± 14	< CCβ	< CCβ	77 ± 14
44	maize silage	409 ± 82	n.d.	n.d.	< CCβ	< CCβ	< CCβ	67 ± 12
45	maize silage	3177 ± 413	n.d.	n.d.	< CCβ	< CCβ	n.d.	704 ± 148
46	maize silage	2247 ± 449	n.d.	n.d.	103 ± 12	n.d.	< CCβ	408 ± 41
47	maize silage	349 ± 63	n.d.	n.d.	< CCβ	< CCβ	< CCβ	271 ± 60
48	maize silage	265 ± 48	n.d.	n.d.	< CCβ	< CCβ	< CCβ	519 ± 93

n.d.= not detected; CCβ= detection capability; DON = deoxynivalenol; DON3G = deoxynivalenol-3-glucoside; DOM-1 = deepoxy-deoxynivalenol; 3-AcDON = 3-acetyl-deoxynivalenol; 15-AcDON = 15-acetyl-deoxynivalenol; β-ZEL = β-zearalenol; α-ZEL = α-zearalenol; ZEN = zearalenone

1. Rasmussen, R.R.; Storm, I.M.L.D.; Rasmussen, P.H.; Smedsgaard, J.; Nielsen, K.F. Multi-mycotoxin analysis of maize silage by LC-MS/MS. *Anal. Bioanal. Chem.* **2010**, *397*, 765–776.
2. Van Pamel, E.; Verbeken, A.; Vlaemynck, G.; de Boever, J.; Daeseleire, E. Ultrahigh-performance liquid chromatographic-tandem mass spectrometric multimycotoxin method for quantitating 26 mycotoxins in maize silage. *J. Agric. Food Chem.* **2011**, *59*, 9747–9755.

3. Dzuman, Z.; Zachariasova, M.; Lacina, O.; Veprikova, Z.; Slavikova, P.; Hajslova, J. A rugged high-throughput analytical approach for the determination and quantification of multiple mycotoxins in complex feed matrices. *Talanta* **2014**, *121*, 263–272.
4. Dagnac, T.; Latorre, A.; Fernández Lorenzo, B.; Llompart, M. Validation and application of a liquid chromatography-tandem mass spectrometry based method for the assessment of the co-occurrence of mycotoxins in maize silages from dairy farms in NW Spain. *Food Addit. Contam. Part A* **2016**, *33*, 1850–1863.
